# Qualitative studies involving users of clinical neurotechnology: a scoping review

**DOI:** 10.1186/s12910-024-01087-z

**Published:** 2024-08-14

**Authors:** Georg Starke, Tugba Basaran Akmazoglu, Annalisa Colucci, Mareike Vermehren, Amanda van Beinum, Maria Buthut, Surjo R. Soekadar, Christoph Bublitz, Jennifer A. Chandler, Marcello Ienca

**Affiliations:** 1 Faculty of Medicine, Institute for History and Ethics of Medicine, Technical University of Munich, Munich, Germany; 2 College of Humanities, École Polytechnique Fédérale de Lausanne, Lausanne, Switzerland; 3 Faculty of Law, University of Ottawa, Ottawa, ON Canada; 4 Clinical Neurotechnology Laboratory, Department of Psychiatry and Neurosciences at the Charité Campus Mitte, Charité – Universitätsmedizin Berlin, Berlin, Germany; 5 Centre for Health Law Policy and Ethics, University of Ottawa, Ottawa, ON Canada; 6 Bertram Loeb Research Chair, Faculty of Law, University of Ottawa, Ottawa, ON Canada; 7 Faculty of Law, Universität Hamburg, Hamburg, Germany

**Keywords:** Neurotechnology, Qualitative research, Subjective experience, Self-perception, Patient-centred technology, Ethics

## Abstract

**Background:**

The rise of a new generation of intelligent neuroprostheses, brain-computer interfaces (BCI) and adaptive closed-loop brain stimulation devices hastens the clinical deployment of neurotechnologies to treat neurological and neuropsychiatric disorders. However, it remains unclear how these nascent technologies may impact the subjective experience of their users. To inform this debate, it is crucial to have a solid understanding how more established current technologies already affect their users. In recent years, researchers have used qualitative research methods to explore the subjective experience of individuals who become users of clinical neurotechnology. Yet, a synthesis of these more recent findings focusing on qualitative methods is still lacking.

**Methods:**

To address this gap in the literature, we systematically searched five databases for original research articles that investigated subjective experiences of persons using or receiving neuroprosthetics, BCIs or neuromodulation with qualitative interviews and raised normative questions.

**Results:**

36 research articles were included and analysed using qualitative content analysis. Our findings synthesise the current scientific literature and reveal a pronounced focus on usability and other technical aspects of user experience. In parallel, they highlight a relative neglect of considerations regarding agency, self-perception, personal identity and subjective experience.

**Conclusions:**

Our synthesis of the existing qualitative literature on clinical neurotechnology highlights the need to expand the current methodological focus as to investigate also non-technical aspects of user experience. Given the critical role considerations of agency, self-perception and personal identity play in assessing the ethical and legal significance of these technologies, our findings reveal a critical gap in the existing literature. This review provides a comprehensive synthesis of the current qualitative research landscape on neurotechnology and the limitations thereof. These findings can inform researchers on how to study the subjective experience of neurotechnology users more holistically and build patient-centred neurotechnology.

**Supplementary Information:**

The online version contains supplementary material available at 10.1186/s12910-024-01087-z.

## Introduction

Due to a rapid expansion in public-private investment, market size and availability of Artificial Intelligence (AI) tools for functional optimization, the clinical advancement of novel neurotechnologies is accelerating its pace [1]. Bidirectional intelligent Brain-Computer interfaces (BCI) that aim at merging both *read-out* and *write-in* devices are in active development and are expanding in functional capabilities and commercial availability. [2, 3]. Such BCIs that can decode and modulate neural activity through direct stimulation of brain tissue, promise additional avenues in the treatment of neurological diseases by adapting to the particularities of individual users’ brain. Potential applications are Parkinson’s disease [4] or epilepsy [5] as well as psychiatric disorders, such as major depressive disorder [6] or obsessive compulsive disorder [7]. Driven by these advances and in conjunction with progress in deep learning and generative AI software as well as higher-bandwidth hardware, clinical neurotechnology is likely to take an increasingly central role in the prevention, diagnosis and treatment of neuropsychiatric disorders.

In line with these scientific trends, the last decade has seen a consequent fast rise in the ethical attention devoted to neurotechnological systems that establish a direct connection with the human central nervous system [8], including neurostimulation devices. Yet, at times, neuroethical concerns may have outpaced real-life possibilities, particularly with view to the impact of neurotechnology on personality, identity, autonomy, authenticity, agency or self (PIAAAS) [9]. This points to the need for basing ethical assessments and personal decisions about deploying devices on solid empirical grounds. In particular, it is crucial to gain a comprehensive understanding of the lived experience of using neurotechnologies from the epistemically privileged first-person perspective of users – “what it is like” to use neurotechnologies. Its examination by empirical studies have added a vital contribution to the literature [10].

Yet, few reviews have attempted to synthesize the growing body of empirical studies on user experience with clinical neurotechnology. Burwell et al. [11] reviewed literature from biomedical ethics on BCIs up to 2016, identifying key ethical, legal and societal challenges, yet noting a lack of concrete ethical recommendations for implementation. Worries about a lack of attention to ethics in BCI studies have been further corroborated by two reviews by Specker Sullivan and Illes, reviewing BCI research published up until 2015. They critically assessed the rationales of BCI research studies [12] and found a remarkable absence of ethical language in published BCI research [13]. Taking a different focus, Kögel et al. [14] have provided a scoping review summarizing empirical studies investigating ethics of BCIs until 2017, with a strong focus on quantitative methods in the reviewed papers. Most recently, this list of reviews has been complemented by van Velthoven et al. [15], who review empirical and conceptual ethical literature on the use of visual neuroprostheses.

To the best of our knowledge, a specific review of qualitative research on the ethics of emerging neurotechnologies such as neuroprosthetics, BCIs and neuromodulation systems is outstanding. We believe that qualitative research involving actual or prospective neurotechnology users is particularly significant as it allows researchers to tap into the richness of first-person experiences as compared to standardized questionnaires without the option of free report. In the following, we synthesize published research on the subjective experience of using clinical neurotechnologies to enrich the ethical debate and provide guidance to developers and regulators.

## Methods

On January 13, 2022 we conducted a search of relevant scientific literature across 5 databases, namely Pubmed (89 results), Scopus (178 results), Web of Science (79 results), PsycInfo (134 results) and IEEE Xplore (4 results). The search was performed for title, abstract and keywords, using a search string to identify articles employing qualitative methods that engaged with users of neurotechnology, and covered normative issues: [“qualitative” OR “interview” OR “focus group” OR “ethnography” OR “grounded theory” OR “discourse analysis” OR “interpretative phenomenological analysis” OR “thematic analysis”] AND [“user” OR “patient” OR “people” OR “person” OR “participant” OR “subject”] AND [“Brain-Computer” OR “BCI” OR “Brain-Machine” OR “neurostimulation” OR “neuromodulation” OR “TMS” OR “transcranial” OR “neuroprosthetic*” OR “neuroprosthesis” OR “DBS”] AND [“ethic*” OR “bioethic*” OR “normative” OR “value” OR “evaluation”].

Across databases, search syntax was adapted to reflect the respective logic of each library. Our search yielded a total of 484 articles. Of these, 133 duplicates were removed. 52 further results were marked as ineligible by automation tools, due to either not being written in English or not representing original research in a peer-reviewed journal. The remaining 299 were screened manually, with screening tasks being shared equally among the authors GS, TBA, AC, MV, CB, JC, and MI. Articles were included if they were written in English, published in a peer-reviewed journal, and reported original research of empirical qualitative findings among human users of a neurotechnological system that establishes a direct connection with the human central nervous system (including neurostimulation devices). Other types of articles such as perspectives, letters to the editor, or review articles were not included. Potential methods included individual interviews, focus groups, stakeholder consultations but excluded studies that did not use any direct verbal input from the users. Each abstract was screened individually by two reviewers. Unclear cases were resolved by discussion among reviewers. This process resulted in the exclusion of 247 articles, leaving 52 publications for inclusion into the final synthesis.

Full texts of these 52 articles were retrieved and assessed for eligibility. Again, this task was shared equally across the 7 authors who made independent recommendations whether an article was included for further analysis, and disagreement was resolved by discussion. 20 articles were excluded at this stage, due to not meeting the inclusion criteria. This resulted in a body of 32 articles plus 4 additional papers identified through citation chaining, as customary in scoping reviews.

In the data analysis phase, we compiled a descriptive summary of the findings and conducted a thematic analysis. When compiling the descriptive summary, we followed the recommendations by Arksey and O’Malley [16] and included comprehensive information beyond authors, year, and title of the study, extracting also study location, methodology, study population, type of neurotechnology, and more. For the thematic analysis, the full text was read and coded by the authors through annotations in pdf files, with papers evenly distributed among the group. Coding was based on a previously agreed coding structure of four thematic families, covering (1) subjective experience with BCIs, (2) aspects concerning usability and technology, (3) ethical questions, (4) impact on social relations, and a fifth miscellaneous category for future resolution. In accordance with the suggestions by Braun and Clarke [17], codes that were not clearly covered by the coding tree were grouped into a category “miscellaneous”, and after discussion used to develop new themes or subsumed under the existing thematic families. The results were compiled and unified by the first author and imported into the Atlas.ti software (version 22.2), with adaptations to the coding tree being discussed between first and last author.

**Fig. 1:**
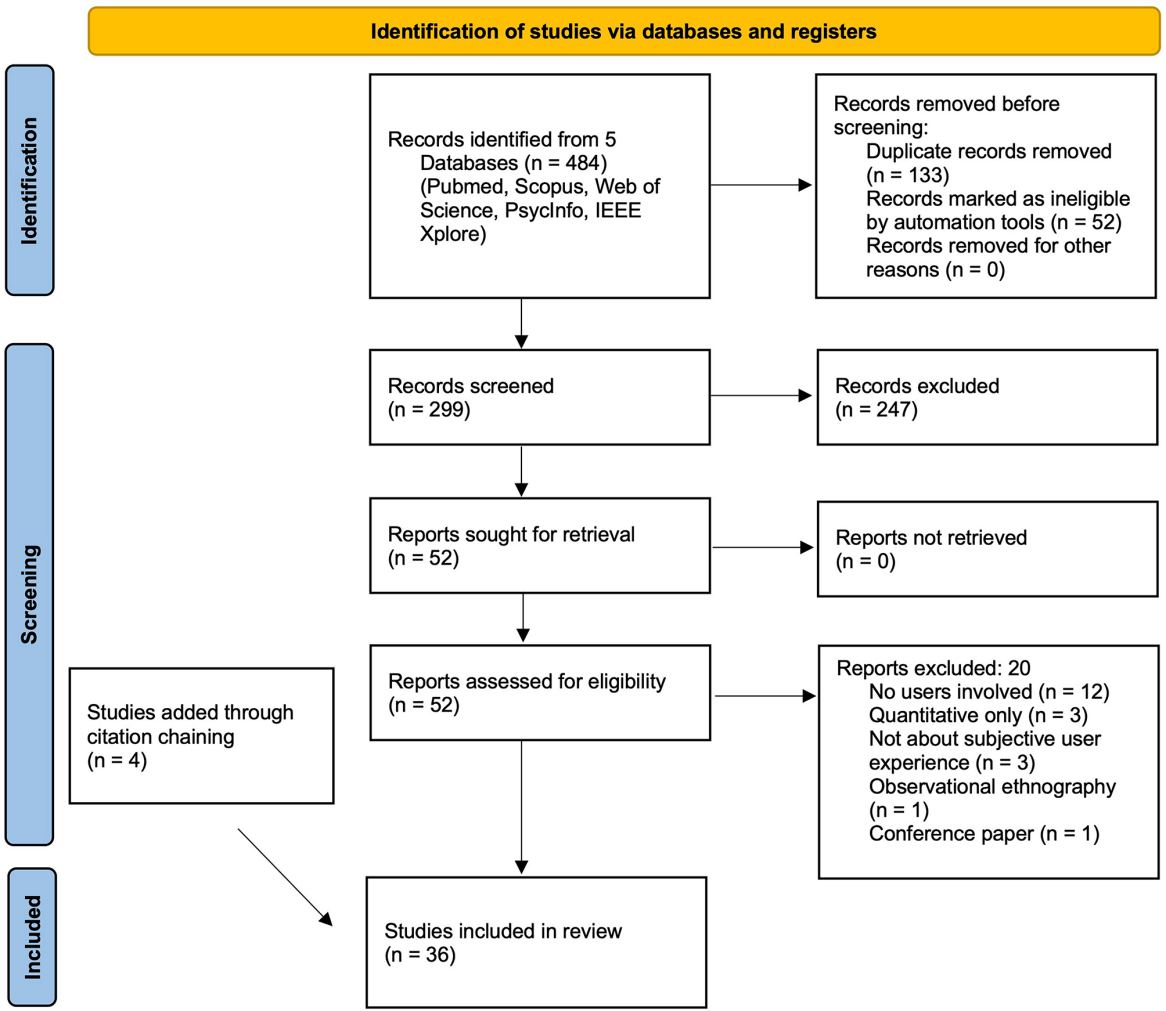
PRISMA flow diagram: search and screening strategy. Based on Page et al [19]

In line with the framework suggested by Pham, Rajić [18], we adhered to the Preferred Reporting Items for Systematic Reviews and Meta-Analyses (PRISMA) in conducting and presenting our results [19]. A flow diagram representing the entire process is depicted in Fig. 1.

## Results

### Descriptive findings

**Fig. 2:**
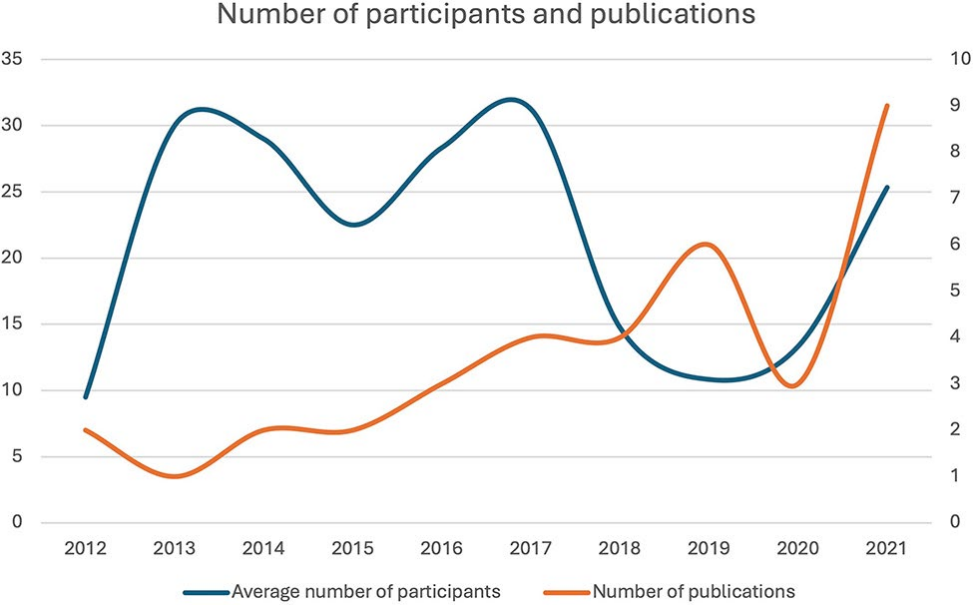
Average number of participants and number of publications over time

Our study included 36 papers reporting original qualitative research among users of BCIs, neuroprosthetics and neuromodulation. We found a pronounced increase in the number of publications employing qualitative methods in the investigation of such neurotechnology users over time, with the earliest study dating back to 2012. However, contrary to what one may expect as reflection of the growing number of neurotechnology users, we did not find an increase in the average sample size of participants enrolled in qualitative studies nor a correlation between year of publication and number of participants (see Fig. 2).

The included studies were exclusively conducted in Western countries, with 11 studies from the US, 9 from Australia and the remaining 16 distributed across Europe (UK: 6, Germany: 4, Sweden, Netherlands and Switzerland 2 each). The majority of studies investigated the effects of invasive neurotechnology in the form of Deep Brain Stimulators (DBS) (26/36), especially in patients with Parkinson’s Disease (PD) (19/36). Many papers also investigated users’ experiences with non-invasive EEG-based BCIs (7/36), whereas all other technologies such as TMS, ECT, FES, intracortical microelectrode arrays, or spinal cord stimulation were only covered by one or two papers each.^1^ Due to the large focus on PD patients, other potential fields for clinical neurotechnological applications were much less present in the analysed research, with only 4 papers each investigating the effects of DBS on patients with major depressive disorder (4/36) or obsessive-compulsive disorder (OCD) (4/36). Across all technologies and patient groups, studies most frequently relied on semi-structured interviews with individual participants (28/36), with much fewer studies using focus groups (3/36) or other qualitative methods.

We found that a large number of papers (14/36) incorporated longitudinal aspects in their study design. With view to non-invasive BCIs, this comprised involving users in the development and testing of BCIs for acquired brain injury [20, 21], assessing subjective reports across sessions for experimental BCI training [22], or having a 2-month follow-up interview for users of a BCI for pain management after spinal cord injury [23]. Studies of invasive devices often included interviews pre- and post-implantation, with a potential third follow-up. In studies with two interviews, the first interview after implantation took place a few weeks after implantation [24, 25], after 3 months [26], after 9 months [27, 28] or after a year [29]. In studies with 3 interviews, post-implantation interviews were either conducted after surgery and again after 3 months in a study on spinal cord stimulation [30] or, in the case of DBS for PD, after 3 and 6 months [31, 32] or after 3–6 and 9–12 months respectively [33]. Table 1 provides a full overview over the included studies.

**Table 1:** Overview over the included studies, reporting involved patient groups, sample size, method, country, investigated neurotechnology and whether invasive or non-invasive technology was investigated

Year	Authors	Title	Patient group	*n*	Method	Country	Neurotechnology	Quality measure		I/NI		
2012	Blain-Moraes et al.	Barriers to and mediators of brain-computer interface user acceptance: Focus group findings	ALS	8	Focus group	US	EEG-based BCI	–		NI		
2012	Mulvenna et al.	Realistic expectations with brain computer interfaces	Acquired brain injury	11	Interviews	UK	EEG-based BCI			NI		
2013	Maier et al.	Patients’ expectations of deep brain stimulation, and subjective perceived outcome related to clinical measures in Parkinson’s disease: a mixed-method approach	PD	30	Semi-structured interview	D	DBS	–		I		
2014	Grubler et al.	Psychosocial and Ethical Aspects in Non-Invasive EEG-Based BCI Research-A Survey Among BCI Users and BCI Professionals	Stroke	19	Semi-structured interview	D/CH	EEG-based BCI	two coding done & checked by two researchers		NI		
2014	Hariz, G., & Hamberg, K.	Perceptions of living with a device-based treatment: An account of patients treated with deep brain stimulation for parkinson’s disease	PD	39	Semi-structured interview	SE	DBS	No COREQ but rigorous description of grounded theory approach		I		
2015	de Haan et al.	Effects of deep brain stimulation on the lived experience of obsessive-compulsive disorder patients: In-depth interviews with 18 patients	OCD	18	Semi-structured interview	NL	DBS	COREQ		I		
2015	Lewis et al.	Subjectively perceived personality and mood changes associated with subthalamic stimulation in patients with Parkinson’s disease	PD	27	Semi-structured interview	D	DBS	–		I		
2016	Hariz, G.M., Limousin, P, & Hamberg, K.	“DBS means everything-For some time”. Patients’ perspectives on daily life with deep brain stimulation for Parkinson’s disease	PD	42	Semi-structured interview	SE	DBS	No COREQ but rigorous description of grounded theory approach		I		
2016	Klein et al.	Brain-computer interface-based control of closed-loop brain stimulation: attitudes and ethical considerations	OCD / Depression	15	Focus group (8), semi-structured interview (7)	US	DBS		–		I	
2016	Maier et al.	Subjective perceived outcome of subthalamic deep brain stimulation in Parkinson’s disease one year after surgery	PD	28	Semi-structured interview	D	DBS		–		I	
2017	de Haan et al.	Becoming more oneself? Changes in personality following DBS treatment for psychiatric disorders: Experiences of OCD patients and general considerations	OCD	18	Semi-structured interview	NL	DBS		–		I	
2017	Kryger et al.	Flight simulation using a Brain-Computer Interface: A pilot, pilot study	Spinocerebellar degeneration	1	Participant’s description of experience during experiment	US	invasive BCI (intracortical microelectrode arrays)		–		I	
2017	LaHue et al.	Parkinson’s disease patient preference and experience with various methods of DBS lead placement	PD	89	Structured interview	US	DBS		–		I	
2017	Gilbert et al.	I Miss Being Me: Phenomenological Effects of Deep Brain Stimulation	PD	17	Semi-structured interview	AUS	DBS		–		I	
2018	Bosanac et al.	Identity challenges and ‘burden of normality’ after DBS for severe OCD: a narrative case study	OCD	1	Narrative analysis	AUS	DBS		COREQ		I	
2018	Gilbert, F., & Viaña, J. N.	A Personal Narrative on Living and Dealing with Psychiatric Symptoms after DBS Surgery	PD	1	Personal narrative	AUS	DBS		–		I	
2018	Kubu et al.	Patients’ shifting goals for deep brain stimulation and informed consent	PD	52	Semi-structured interview	US	DBS		No COREQ but rigorous description of grounded theory approach		I	
2018	Martin et al.	A qualitative study adopting a user-centered approach to design and validate a brain computer interface for cognitive rehabilitation for people with brain injury	Traumatic brain injury	5	Oral feedback at the end of experiment	UK	EEG-based BCI				NI	
2019	Al-Taleb et al.	Home used, patient self-managed, brain-computer interface for the management of central neuropathic pain post spinal cord injury: Usability study	Central neuropathic pain in people with spinal cord injury.	15	Semi-structured interview	UK	EEG-based neurofeedback		–		NI	
2019	Gilbert et al.	Embodiment and Estrangement: Results from a First-in-Human “Intelligent BCI” Trial	Epilepsy	6	Semi-structured interview	AUS	Intelligent implantable BCI (predictive & advisory function); intracranial electrodes on cortical surface		–		I	
2019	Liddle et al.	Impact of deep brain stimulation on people with Parkinson’s disease: A mixed methods feasibility study exploring lifespace and community outcomes	PD	8	Semi-structured interview	AUS	DBS		No COREQ but mixed methods quality checks such as triangulation or following themes from one data type to the other		I	
2019	Liddle et al.	Mapping the experiences and needs of deep brain stimulation for people with Parkinson’s disease and their family members	PD	14	Semi-structured interview	AUS	DBS		No Coreq but lengthy description of coding process		I	
2019	Ryan et al.	An Exploration of the Experiences and Educational Needs of Patients With Failed Back Surgery Syndrome Receiving Spinal Cord Stimulation	Failed back surgery syndrom	12	Semi-structured interview	UK	Spinal Cord Stimulation		–		I	
2019	Shahmoon, S.; Smith, J. A.; Jahanshahi, M.	The Lived Experiences of Deep Brain Stimulation in Parkinson’s Disease: An Interpretative Phenomenological Analysis	PD	10	Semi-structured interview	UK	DBS				I	
2020	Cabrera, L. Y.; Kelly-Blake, K.; Sidiropoulos, C.	Perspectives on Deep Brain Stimulation and Its Earlier Use for Parkinson’s Disease: A Qualitative Study of US Patients	PD	20	(semi-)structured interview	US	DBS		–		I	
2020	Kögel, J.; Jox, R. J.; Friedrich, O.	What is it like to use a BCI? - insights from an interview study with brain-computer interface users	different muscular conditions	9	Semi-structured interview	D	invasive & non-invasive (EEG-based) BCIs (active or reactive; no passive such as DBS)		Comprehensive description of grounded theory approach		NI & I	
2020	Thomson et al.	“He’s back so I’m not alone”: The impact of deep brain stimulation on personality, self, and relationships in Parkinson’s disease	PD	11	Semi-structured interview	AUS	DBS		crosscoding; no COREQ		I	
2021	Chacón Gámez, Y. M.; Brugger, F.; Biller-Andorno, N.	Parkinson’s Disease and Deep Brain Stimulation Have an Impact on My Life: A Multimodal Study on the Experiences of Patients and Family Caregivers	PD	44	Semi-structured interview	CH	DBS		report on researchers’ reflexivity; double-coding; coding tree checked with further team members		I	
2021	Merner et al.	Changes in Patients’ Desired Control of Their Deep Brain Stimulation and Subjective Global Control Over the Course of Deep Brain Stimulation	PD	52	Semi-structured interview	US	DBS		–		I	
2021	Mosley et al.	‘Woe Betides Anybody Who Tries to Turn me Down.’ A Qualitative Analysis of Neuropsychiatric Symptoms Following Subthalamic Deep Brain Stimulation for Parkinson’s Disease	PD	10	Semi-structured interview	AUS	DBS		COREQ		I	
2021	Bluhm et al.	They Affect the Person, but for Better or Worse? Perceptions of Electroceutical Interventions for Depression Among Psychiatrists, Patients, and the Public	Depression	48	Semi-structured interview	US	ECT; TMS; DBS		–		I & NI	
2021	Sankary et al.	Exit from Brain Device Research: A Modified Grounded Theory Study of Researcher Obligations and Participant Experiences	Stroke, Depression, Epilepsy	16	Semi-structured interview	US	Investigational brain implants (DBS, responsive neurostimulation)		Discussion of data saturation (Corbin & Strauss)		I	
2021	Thomson et al.	“Nothing to Lose, Absolutely Everything to Gain”: Patient and Caregiver Expectations and Subjective Outcomes of Deep Brain Stimulation for Treatment-Resistant Depression	Depression	6	Semi-structured interview	AUS	DBS		COREQ		I	
2021	Zulauf-Czaja et al.	On the way home: a BCI-FES hand therapy self-managed by sub-acute SCI participants and their caregivers: a usability study	Spinal cord injury (tetraplegic)	8	Focus group & interview	UK	EEG & functional electrical stimulation		–		NI	
2021	Wexler et al.	Ethical Issues in Intraoperative Neuroscience Research: Assessing Subjects’ Recall of Informed Consent and Motivations for Participation	PD	22	Semi-structured interview	US	DBS		-		I	
2021	Goering, S., Wexler, A. and Klein, E.	Trading Vulnerabilities: Living with Parkinson’s Disease before and after Deep Brain Stimulation	PD	22	Semi-structured interview	US	DBS		-		I	

### Thematic findings

Our findings from the thematic analysis can be grouped into four overlapping thematic families, namely (1) ethical challenges of neurotechnology use, (2) subjective experience with clinical neurotechnologies, (3) impact on social relations, and (4) usability and technological aspects. The raw data of our findings are accessible in the supplementary file.

### Ethical concerns

With respect to users’ experiences of neurotechnology that touch on classical ethical topics, we found that autonomy played a central role in slightly more than half of all papers (20/36), yet in four different ways. Many papers noted the positive impact neurotechnology has on users’ autonomy. Users often perceive the technology as enabler of greater control over their own life, allowing them “to become who they wanted to be” [2], providing them with agency and greater independence, restoring their ability to help others, or allowing them to be more spontaneous in their everyday life [2, 10, 28, 31, 32, 34–37]. Some studies reported how neurotechnology may impact users’ autonomy negatively, especially by making them more dependent on technological and medical support [25, 28, 35, 38, 39]. When balancing these positive and negative impacts, some users seem to prefer such dependency and to leave control over the devices to healthcare professionals, to ensure its safe and appropriate working [2, 32, 39, 40]. Also related to autonomy were concerns about consent, especially with a view to the level of information patients received before the implantation of an invasive device, which was deemed inadequate by some patients [2, 24, 31, 34, 38–46]. Several papers called to include patients during the technology design process [2, 31, 39]. In addition, questions of responsibility and accountability in case of malfunctioning were repeatedly named as key concern [10, 25, 37, 38, 45, 47].

Concerns about beneficence and about harming patients also featured prominently in most of the analysed papers (24/36), yet with substantive differences on a more granular level. While symptom improvement and restorative changes were widely reported [2, 10, 23, 26, 29, 31, 33–35, 38–40, 43, 44, 46], some users reported experiencing physical or psychological side effects, such as postoperative complications, new worries – for instance about magnetic fields or about changing batteries –, stigma, or becoming more aware of their past suffering [23, 25, 26, 28, 34–40, 42, 46, 48, 49]. Less frequently we found concerns about patient-doctor-relationships [2, 24, 32, 40, 42, 43], which seem to mediate the acceptance of clinical neurotechnologies but are also themselves impacted by technology use. For instance, while some research points to the importance of patients’ trust in healthcare professionals for the acceptance of neurotechnology [24], a personal narrative described a breakdown of patient-physician relationship following a distressful DBS implantation for treating PD [42].

### Impact on subjective experiences

Since the subjective lived experiences of neurotechnology users commonly constituted the central element of the reviewed qualitative papers, we found a rich field of reports in the vast majority of paper (31/36), describing experiences that were perceived as positive, negative or neutral. Neurotechnology-induced behavioural changes [28, 36, 37, 40, 42, 46, 47, 49], as well as changes in feelings [27, 41, 42], (self-) perception [10, 23, 34, 36, 40–42, 44, 48, 50], personality [27, 29, 34–37, 42–44, 47, 49], preferences [49, 50] or thinking [10, 41] were also reported, particularly in users receiving continuous, non-adaptive deep brain stimulation (DBS).

Behavioural changes often concerned desired outcomes such as fewer obsessive thoughts and compulsive behaviours after successful OCD treatment [49], acting with less impediment due to seizure predictions [36], or acting more boldly with more energy and increased confidence due to symptom improvement in PD [37, 47]. Nevertheless, it was necessary for patients and for their environment to adapt and get used to new patterns of behaviour. Some patients also reported undesirable behavioural changes after subthalamic DBS implantation, “bordering on mania” [42], such as being excessively talkative [46] or shopping compulsions that were later described by the patient as “ridiculous” [28].

These outwardly observable changes were often related to psychological changes that users reported. Some DBS users experienced mood changes, ranging from elevated to depressed [27, 41, 42, 44], while others reported changed preferences. Sometimes this affected what users valued as important in life [50], sometimes it related to very particular preferences, such as taste in music, with one patient attributing a transition from The Rolling Stones and The Beatles to Johnny Cash to their DBS implantation [49]. In patients treated for OCD or motor disorders, two studies also found positive impact on users’ thinking, whether by freeing them from obsessive thoughts [41] or improving their concentration skill [10]. In line with the large neuroethical debate on the subject, changes at times amounted to what neurotechnology users described as personality changes. Such changes included negative impacts such as being more irritable, anxious or less patient [34, 35] or overly increased libido [49], neutral changes, such as (re-)taking an interest in politics or movies [49], and positive changes linked to improvement of psychiatric symptoms, such as being more easy-going and daring, being more expressive and assertive, or simply being more confident [35, 49].

**Fig. 3:**
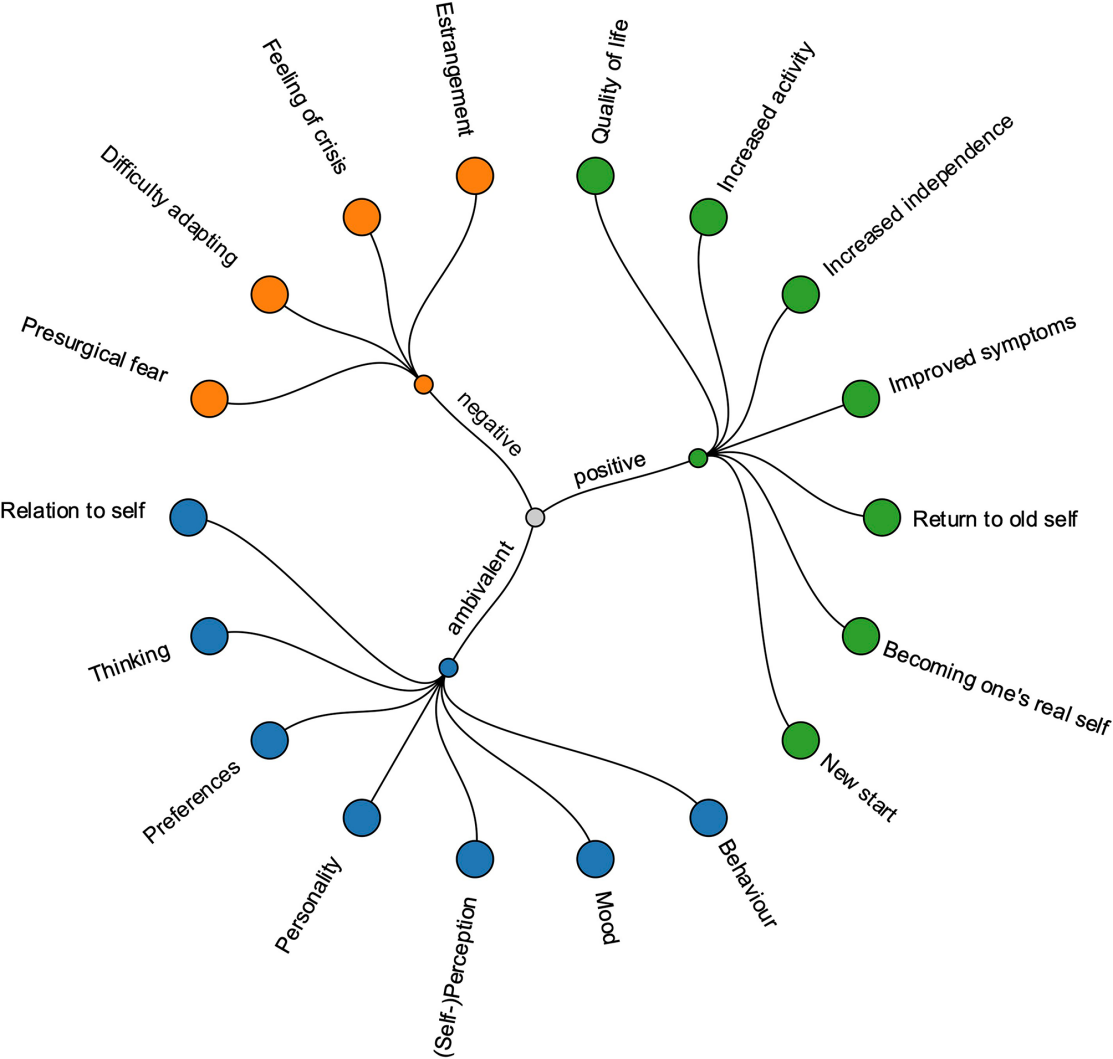
Impact of clinical neurotechnology on subjective experience. The colours represent the valence of the impact, with orange dots representing negative, green dots representing positive, and blue dots representing ambivalent changes

In line with the diversity of these changes, patients reported a vast spectrum of different attitudes towards and relations with the neurotechnology. Some users embraced the BCI explicitly as part of themselves [14, 37, 39, 49] and described how “DBS becomes a part of who you are rather than changing you” [37]. Others felt estranged using the BCI [28, 36, 37, 42, 49] and even expressed desires to remove the alien device in forceful terms: “I hate it! I wish I could pull it out!” [37]. Aside from changes brought about by the device, the patients’ state before using neurotechnology and especially their relation to their illness seemed to play a crucial role [28, 51]. An overview over the different thematic findings is provided in Fig. 3.

The overwhelming majority of studies (23/36) reported improvements of the treated symptoms [2, 26, 28, 31, 33–35, 37, 40–43, 46–50, 52], making patients’ lives easier [48, 49] or – as some put it – even saving their lives [34, 45, 48]. Patients felt that the neurotechnology allowed them an increase in activity [33, 34, 40] and a return to previous forms of behaviour [33, 40, 48, 49], strengthening their sense of freedom and independence [2, 10, 22, 33–36, 40, 43, 49, 50, 53]. Emotionally, users reported feeling more daring [29, 35], self-confident [28, 35–37, 44] or more stable [34, 50] as well as feelings of hope or joy [10, 22, 35, 50]. For better or worse, such changes were sometimes perceived as providing a “new start” [34, 48] or even a “new identity” [34, 41, 42, 49], while others perceived their changes as a reversion to their “former” [28, 29, 47, 49, 50] or their “real” self [36, 42, 49].

Among the negative subjective impacts of clinical neurotechnology mentioned in the literature (16/36), users commonly reported issues of estrangement, caused by self-perceived changes to behaviour, feelings, personality traits, or patients’ relation to their disease or disorder [28, 36, 37, 42, 49]. The negative impact differed largely depending on the type of neurotechnology used as well as on the disorders and symptoms treated with the technology. While ALS patients as users of non-invasive BCIs for spelling interfaces reported increased anxiety in interaction with the devices [53], PD patients with invasive DBS reported presurgical fears of pain and of the invasive procedure as well as fear of outward manipulation within their brain through the DBS implantation [40, 43, 54]. Frequently, it was not entirely clear whether adverse developments such as further cognitive decline were attributable to the implanted device or to the persisting disease and its natural trajectory [31, 33, 34, 40, 43, 48, 50]. However, occasionally very severe psychiatric consequences of treatment were reported, notably by one PD patient who experienced mania and depressive symptoms through DBS treatment, resulting in a suicide attempt [42]. For DBS patients with OCD, negative impacts seem more related to difficulties of adapting to the new situation [35, 49], for instance to their suddenly increased libido as a side-effect of DBS use that may be perceived as “too much” [49], or to a perceived lack of preparation for their new (OCD-free) identity [41]. In two studies on patients with OCD, the sudden improvement of symptoms also led to moments of existential crisis, given that the symptoms had shaped a great part of their previous daily activities [41, 49].

### Impact on social relations

Using a neurotechnology not only impacts users but can also affect social relations with others (23/36), particularly primary caregivers. While some neurotechnologies such as non-invasive BCIs for communication may create additional workload for caregivers if the BCI needs to be set up, neurotechnologies can also reduce their burden by rendering patients more independent [10, 34, 40, 53]. Beyond workload, neurotechnologies were also reported to enrich social relations by facilitating communication [10, 34, 53], though in some cases, they led to potential tension between informal caregivers and patients, e.g. due to personality changes [28, 35, 37, 40, 42, 47, 49, 55] or if the device was blamed for a patient’s behaviour or suggested as a solution to interpersonal problems [2]. Whether positive or negative, family and social support were reportedly playing a vital role in the treatment [2, 28, 40, 50].

Similarly important was support by clinicians [39, 40] and the wish for support groups with fellow neurotechnology users [27, 30, 40, 41]. Inclusion in research activities was also reported as a positive effect of (experimental) BCIs [10, 38]. More importantly though, in a large number of studies, neurotechnology users reported positive effects on their social relations [2, 29, 35, 43, 46, 48, 50], with some users reporting an increased wish to help others [35, 50]. A negative social consequence in public was perceived stigma [25, 35, 48], even though some patients chose to actively show their device in public, “to spread information and knowledge about this treatment” [39].

### Usability concerns

Concerns with technical questions and usability issues comprising efficiency, effectiveness and satisfaction [52] were also raised by almost half of the research papers (17/36), yet differed greatly between neurotechnologies, owing to large differences in hardware (e.g., between EEG caps and implanted electrodes) and handling (e.g., between passive neurostimulation or training-intensive active BCIs). Across all applications, invasive as much as non-invasive, the most frequent concerns (8/36 each) related to hardware issues [2, 22, 23, 38, 39, 46, 52, 53] as well as to the required fine-tuning of devices to find optimal settings, associated with time-burden for their users [20, 23, 27, 32, 39, 46, 50, 56]. Similarly, the training of patients required for the successful use of non-invasive, active BCIs was reported as being perceived as cumbersome or complicated, providing a potential obstacle to their implementation in everyday contexts [38, 52]. Several studies reported that the use of such active BCIs required considerable concentration, leading to fatigue after prolonged use [10, 38, 53]. Mediating factors to address such obstacles were the availability of technical support [33, 53], general attitudes towards technology [53], ease of integrating the technology into everyday life [10, 38, 53] and realistic expectations regarding the neurotechnology’s effects [30, 38, 40, 46].

## Discussion

The identified publications highlight that qualitative research through interviews and focus groups offers a useful way to gain access to the subjective experience of users of a diverse range of neurotechnologies. Such investigation of users’ privileged knowledge about novel devices in turn is crucial to improve future neurotechnological developments and align them with ethical considerations already at an early stage [57]. Here, we discuss our findings by comparing different clinical neurotechnologies, identify gaps in the literature and point to the limitations of our scoping review.

One finding of our scoping review is that qualitative research on neurotechnologies has so far primarily focused on users of DBS treated for PD. In part, this may reflect that DBS is an established, effective treatment for controlling motor symptoms in PD, improving patients’ quality of life, resulting in its wide-spread adoption in many different healthcare systems worldwide [58–61]. Still, it would be highly beneficial to extend qualitative research to different patient groups and other clinical neurotechnologies that directly target mental states or processes, where more pronounced effects of subjective experiences may be expected.

A potential obstacle to involving more neurotechnology users beyond PD patients treated with DBS is that, for many other technologies, users are still likely to receive their treatment as part of an experimental trial. Qualitative research with such patients may face the additional practical barrier of convincing the other researchers to facilitate access to their patients. Better communication across disciplines and research fields may facilitate such access, providing much-needed insights into user experiences of experimental neurotechnologies.

Some of the articles reviewed here already offer such perspectives, e.g. the ones investigating DBS used for major depressive disorder or OCD. Such research may also help to further clarify which differences in subjective outcome are owed to technology and which are owed to differences in the treated disorders. As different patient groups are likely to have different needs and views, further research is needed to explore those needs and views and develop implementation strategies designed to address them in a patient-tailored manner. Furthermore, different neurotechnologies (and applications thereof) are likely to impact the mind of their users in a different way. Therefore, future research should investigate whether the type and modality of stimulation exert differential impacts on the subjective experience of the end users.

Our findings reveal differential effects among patients using DBS for the treatment of PD and patients using DBS for the treatment of OCD, respectively. For example, some reported effects of invasive neurotechnology such as the induction of more assertive behaviour may be a reason for concern in PD [28], while being considered a successful treatment outcome in OCD [35, 49]. More comparative research among DBS users treated for OCD or other neuropsychiatric disorders, such as depression, are needed [62] and may help to better understand which experiences are directly attributable to the stimulation of specific brain areas such as the subthalamic nucleus for PD and the nucleus accumbens for OCD, and which result from other factors, e.g., related to undergoing surgery or to different treatment settings in neurological and psychiatric care [63, 64].

Research on such differences may also imply practical consequences. For instance, one may wonder whether different preparation stages and possibly different degrees of information for obtaining consent may be called for between invasive clinical neurotechnologies used in psychiatry and neurology—or whether, on the contrary, similarities in the use of neurotechnologies ultimately point towards ending the distinction between mental and neurological illnesses [63]. In either case, our findings highlight that psychological impacts of clinical neurotechnologies are complex and multi-faceted phenomena—mediated by many factors—calling for more qualitative research to better grasp the lived experiences of those using novel neurotechnologies.

Our scoping review identified several gaps in the literature related to research methodology, investigated topics and investigated neurotechnologies. First, while a large number of studies embrace a longitudinal approach to investigating users’ experiences, none of the included studies looked at impacts beyond a timeframe of one year. However, as is known from DBS studies in major depressive disorder, it is important to investigate and evaluate long-term effects of neurotechnologies such as DBS [6]. Future qualitative research should therefore address this gap. Connected to this are, second, research questions that have not yet been investigated in full, such as long-term impacts of clinical neurotechnologies on memory or belief continuity. Third, empirical findings on closed-loop neurotechnologies that integrate artificial intelligence are so far nascent [2, 36]. As there are important conceptual and ethical questions that arise specifically from the integration of human and artificial intelligence, e.g. questions of control and responsibility, further qualitative research should be conducted on users of such devices.

Finally, our findings reveal a complex and multifaceted landscape of ethical considerations. While considerations regarding personal autonomy appear largely prevalent among users, the perceived or expected impacts of neurotechnology use on personal autonomy differ significantly. Some studies suggest that neurotechnology use may enhance personal autonomy by allowing users to be more autonomous and independent in their daily lives and even restore part of the autonomous control that was disrupted by their disorders. Other studies suggest that some neurotechnologies, especially neural implants relying on autonomous components, may diminish autonomy as they may override some users’ intentions. Sometimes this ambivalent effect is observed within the same study. This is consistent with previous theoretical reflections on this topic [65] and urges scientists to develop fine-grained and patient-centred models for assessing the impact of neurotechnology on personal autonomy. These models should distinguish on-target and off-target effects and elucidate which subcomponents of personal autonomy (e.g., volition, behavioural control, authenticity etc.) are impacted by the use of neurotechnology.

Our scoping review has several limitations. Owing to the nature of a scoping review and to our inclusion criteria, there may be relevant literature that we missed to identify and analyse. For instance, since we only included English publications, we may have missed relevant research published in other languages, which may explain why we only found qualitative studies conducted in Western countries. Furthermore, our narrow search strategy excluded other relevant research, for instance qualitative studies conducted with potential users of clinical neurotechnology or with caregivers. Yet, a scoping reviews can provide a useful tool to map existing literature [16, 18], and given recent advances in technology and accompanying qualitative research, an update of earlier reviews such as the one by Kögel et al. [14], provides an important addition to the existing literature. By looking at qualitative studies only we further import general limitations of qualitative studies, such as a lack of generalizability and a dependency on the skills and experience of the involved researchers. More standardized instruments to complement the investigation of subjective experiences of neurotechnology users therefore seem highly desirable. Recent quantitative approaches such as online surveys assessing the subjective preferences of DBS users concerning the timing of implantation [66] or studies combining qualitative data with quantitative assessments [67] point in this direction. Additionally, experimental approaches to the monitoring and evaluation of the effects of neurotechnology on the user’s experience are currently absent. Therefore, future research should complement qualitative and quantitative user evaluations based on social science methods (e.g., interviews, focus groups and questionnaires) with experimental models.

## Conclusion

The findings of our review emphasize the diversity of individual experiences with neurotechnology across individuals and different technologies. They underscore the need to conduct qualitative research among diverse groups at different time-points to better assess the impact of such technologies on their users, which are important to inform requirements of efficacy and safety for clinical neurotechnologies. In addition, qualitative research offers one way to implement user-centred ethical considerations into product development through user-centred design and to accompany the development of novel neurotechnologies with ethical considerations as they mature and become clinical standard.

### Electronic supplementary material

Below is the link to the electronic supplementary material.

Supplementary Material 1

## Data Availability

The availability of the full data supporting the findings of this study is subject to restrictions due to the copyright of the included papers. The quotes analysed during this study are included in this published article and its supplementary information files. Further data are available from the authors upon request.

## Footnotes

1 As many publications included patients with different diagnoses or investigated the effects of different neurotechnologies, the numbers indicated here do not add up.

## References

[1] UNESCO. Unveiling the neurotechnology landscape: scientific advancements innovations and major trends. 2023.

[2] Klein E, et al. Brain-computer interface-based control of closed-loop brain stimulation: attitudes and ethical considerations. Brain-Computer Interfaces. 2016;3(3):140–8. 10.1080/2326263X.2016.1207497

[3] Kellmeyer P, et al. The effects of closed-loop medical devices on the autonomy and accountability of persons and systems. Camb Q Healthc Ethics. 2016;25(4):623–33. 27634714 10.1017/S0963180116000359

[4] Limousin P, Foltynie T. Long-term outcomes of deep brain stimulation in Parkinson disease. Nat Reviews Neurol. 2019;15(4):234–42. 10.1038/s41582-019-0145-9 30778210

[5] Alkawadri R. Brain–computer interface (BCI) applications in mapping of epileptic brain networks based on intracranial-EEG: an update. Front NeuroSci. 2019;13:191. 30971871 10.3389/fnins.2019.00191 PMC6446441

[6] Crowell AL, et al. Long-term outcomes of subcallosal cingulate deep brain stimulation for treatment-resistant depression. Am J Psychiatry. 2019;176(11):949–56. 31581800 10.1176/appi.ajp.2019.18121427

[7] Mar-Barrutia L, et al. Deep brain stimulation for obsessive-compulsive disorder: a systematic review of worldwide experience after 20 years. World J Psychiatry. 2021;11(9):659. 34631467 10.5498/wjp.v11.i9.659 PMC8474989

[8] Clausen J, et al. Help, hope, and hype: ethical dimensions of neuroprosthetics. Science. 2017;356(6345):1338–9. 28663460 10.1126/science.aam7731

[9] Gilbert F, Viaña JNM, Ineichen C. Deflating the DBS causes personality changes bubble. Neuroethics. 2021;14(1):1–17. 10.1007/s12152-018-9373-8

[10] Kögel J, Jox RJ, Friedrich O. What is it like to use a BCI? - insights from an interview study with brain-computer interface users. BMC Med Ethics. 2020;21(1):2. 31906947 10.1186/s12910-019-0442-2 PMC6945485

[11] Burwell S, Sample M, Racine E. Ethical aspects of brain computer interfaces: a scoping review. BMC Med Ethics. 2017;18(1):1–11. 29121942 10.1186/s12910-017-0220-y PMC5680604

[12] Sullivan LS, Illes J. Beyond ‘communication and control’: towards ethically complete rationales for brain-computer interface research. Brain-Computer Interfaces. 2016;3(3):156–63. 10.1080/2326263X.2016.1213603

[13] Specker Sullivan L, Illes J. Ethics in published brain–computer interface research. J Neural Eng. 2018;15(1):013001. 28931749 10.1088/1741-2552/aa8e05

[14] Kögel J, et al. Using brain-computer interfaces: a scoping review of studies employing social research methods. BMC Med Ethics. 2019;20(1):18. 30845952 10.1186/s12910-019-0354-1 PMC6407281

[15] van Velthoven E, et al. Ethical implications of visual neuroprostheses—a systematic review. J Neural Eng. 2022;19(2):026055. 10.1088/1741-2552/ac65b2 35475424

[16] Arksey H, O’Malley L. Scoping studies: towards a methodological framework. Int J Soc Res Methodol. 2005;8(1):19–32. 10.1080/1364557032000119616

[17] Braun V, Clarke V. Using thematic analysis in psychology. Qualitative Res Psychol. 2006;3(2):77–101. 10.1191/1478088706qp063oa

[18] Pham MT, et al. A scoping review of scoping reviews: advancing the approach and enhancing the consistency. Res Synthesis Methods. 2014;5(4):371–85. 10.1002/jrsm.1123 PMC4491356 26052958

[19] Page MJ, et al. The PRISMA 2020 statement: an updated guideline for reporting systematic reviews. Syst Reviews. 2021;10(1):1–11. 10.1186/s13643-021-01626-4 PMC8008539 33781348

[20] Mulvenna M, et al. Realistic expectations with brain computer interfaces. J Assist Technol. 2012;6(4):233–44. 10.1108/17549451211285735

[21] Martin S, et al. A qualitative study adopting a user-centered approach to design and validate a brain computer interface for cognitive rehabilitation for people with brain injury. Assist Technol. 2018;30(5):233–41. 28708963 10.1080/10400435.2017.1317675

[22] Kryger M, et al. Flight simulation using a brain-computer interface: a pilot, pilot study. Exp Neurol. 2017;287:473–8. 27196543 10.1016/j.expneurol.2016.05.013

[23] Al-Taleb M, et al. Home used, patient self-managed, brain-computer interface for the management of central neuropathic pain post spinal cord injury: usability study. J Neuroeng Rehabil. 2019;16(1):1–24. 31666096 10.1186/s12984-019-0588-7 PMC6822418

[24] Wexler A, et al. Ethical issues in intraoperative neuroscience research: assessing subjects’ recall of informed consent and motivations for participation. AJOB Empir Bioeth. 2022;13(1):57–66. 34227925 10.1080/23294515.2021.1941415 PMC9188847

[25] Goering S, Wexler A, Klein E. Trading vulnerabilities: living with Parkinson’s Disease before and after deep brain stimulation. Camb Q Healthc Ethics. 2021;30(4):623–30. 34702406 10.1017/S0963180121000098 PMC9215176

[26] Maier F, et al. Patients’ expectations of deep brain stimulation, and subjective perceived outcome related to clinical measures in Parkinson’s disease: a mixed-method approach. J Neurol Neurosurg Psychiatry. 2013;84(11):1273–81. 23715910 10.1136/jnnp-2012-303670

[27] Thomson CJ, Segrave RA, Carter A. Changes in Personality Associated with Deep Brain Stimulation: a qualitative evaluation of clinician perspectives. Neuroethics. 2021;14:109–24. 10.1007/s12152-019-09419-2

[28] Thomson CJ, et al. He’s back so I’m not alone: the impact of deep brain stimulation on personality, self, and relationships in Parkinson’s disease. Qual Health Res. 2020;30(14):2217–33. 32856559 10.1177/1049732320951144

[29] Lewis CJ, et al. Subjectively perceived personality and mood changes associated with subthalamic stimulation in patients with Parkinson’s disease. Psychol Med. 2015;45(1):73–85. 25066623 10.1017/S0033291714001081

[30] Ryan CG, et al. An exploration of the experiences and Educational needs of patients with failed back surgery syndrome receiving spinal cord stimulation. Neuromodulation. 2019;22(3):295–301. 30451347 10.1111/ner.12885

[31] Kubu CS, et al. Patients’ shifting goals for deep brain stimulation and informed consent. Neurology. 2018;91(5):e472–8. 29959262 10.1212/WNL.0000000000005917 PMC6093764

[32] Merner AR, et al. Changes in patients’ desired control of their deep brain stimulation and subjective Global Control over the Course of Deep Brain Stimulation. Front Hum Neurosci. 2021;15:642195. 33732125 10.3389/fnhum.2021.642195 PMC7959799

[33] Liddle J, et al. Impact of deep brain stimulation on people with Parkinson’s disease: a mixed methods feasibility study exploring lifespace and community outcomes. Hong Kong J Occup Ther. 2019;32(2):97–107. 32009861 10.1177/1569186119865736 PMC6967222

[34] Chacón Gámez YM, Brugger F, Biller-Andorno N. Parkinson’s Disease and Deep Brain Stimulation Have an Impact on My Life: A Multimodal Study on the Experiences of Patients and Family Caregivers. Int J Environ Res Public Health. 2021;18(18):9516. 10.3390/ijerph18189516 PMC8467519 34574440

[35] de Haan S et al. Effects of deep brain stimulation on the lived experience of obsessive-compulsive disorder patients: in-depth interviews with 18 patients. PLoS One. 2015;10(8):e0135524. 10.1371/journal.pone.0135524 PMC4552296 26312488

[36] Gilbert F, et al. Embodiment and estrangement: results from a first-in-Human Intelligent BCI Trial. Sci Eng Ethics. 2019;25(1):83–96. 29129011 10.1007/s11948-017-0001-5 PMC6418065

[37] Gilbert F, et al. I miss being me: phenomenological effects of deep brain stimulation. AJOB Neurosci. 2017;8(2):96–109. 10.1080/21507740.2017.1320319

[38] Grübler G, et al. Psychosocial and ethical aspects in non-invasive EEG-based BCI research - A survey among BCI users and BCI professionals. Neuroethics. 2014;7(1):29–41. 10.1007/s12152-013-9179-7

[39] Hariz G-M, Hamberg K. Perceptions of living with a device-based treatment: an account of patients treated with deep brain stimulation for Parkinson’s disease. Neuromodulation: Technol Neural Interface. 2014;17(3):272–8. 10.1111/ner.12073 24033886

[40] Liddle J, et al. Mapping the experiences and needs of deep brain stimulation for people with Parkinson’s disease and their family members. Brain Impairment. 2019;20(3):211–25. 10.1017/BrImp.2019.3

[41] Bosanac P, et al. Identity challenges and ‘burden of normality’ after DBS for severe OCD: a narrative case study. BMC Psychiatry. 2018;18(1):186. 29895269 10.1186/s12888-018-1771-2 PMC5998583

[42] Gilbert F, Viaña JN. A personal narrative on living and dealing with Psychiatric symptoms after DBS surgery. Narrat Inq Bioeth. 2018;8(1):67–77. 29657181 10.1353/nib.2018.0024

[43] Cabrera LY, Kelly-Blake K, Sidiropoulos C. Perspectives on deep brain stimulation and its earlier use for parkinson’s disease: a qualitative study of US patients. Brain Sci. 2020;10(1). 10.3390/brainsci10010034 PMC7016580 31936218

[44] Bluhm R, et al. They affect the person, but for Better or worse? Perceptions of Electroceutical interventions for Depression among psychiatrists, patients, and the Public. Qual Health Res. 2021;31(13):2542–53. 34672815 10.1177/10497323211037642 PMC8579329

[45] Sankary LR et al. Exit from Brain Device Research: A Modified Grounded Theory Study of Researcher Obligations and Participant Experiences. AJOB Neurosci. 2021;1–12. 10.1080/21507740.2021.1938293 PMC10570922 34255614

[46] Thomson CJ, et al. Nothing to lose, absolutely everything to Gain: patient and caregiver expectations and subjective outcomes of deep brain stimulation for treatment-resistant depression. Front Hum Neurosci. 2021;15:755276. 34658822 10.3389/fnhum.2021.755276 PMC8511461

[47] Mosley PE, et al. Woe betides anybody who tries to turn me down.’ A qualitative analysis of neuropsychiatric symptoms following subthalamic deep brain stimulation for Parkinson’s Disease. Neuroethics. 2021;14:47–63. 10.1007/s12152-019-09410-x

[48] Hariz G-M, Limousin P, Hamberg K. DBS means everything-for some time. Patients’ perspectives on daily life with deep brain stimulation for Parkinson’s disease. J Parkinson’s Disease. 2016;6(2):335–47. 27003786 10.3233/JPD-160799 PMC4927913

[49] de Haan S, et al. Becoming more oneself? Changes in personality following DBS treatment for psychiatric disorders: experiences of OCD patients and general considerations. PLoS ONE. 2017;12(4):e0175748. 28426824 10.1371/journal.pone.0175748 PMC5398533

[50] Shahmoon S, Smith JA, Jahanshahi M. The lived experiences of deep brain stimulation in parkinson’s disease: an interpretative phenomenological analysis. Parkinson’s Disease. 2019;2019(1):1937235. 10.1155/2019/1937235 PMC6377998 30854185

[51] Adamson AS, Welch HG. Machine learning and the Cancer-diagnosis problem - no gold Standard. N Engl J Med. 2019;381(24):2285–7. 31826337 10.1056/NEJMp1907407

[52] Zulauf-Czaja A, et al. On the way home: a BCI-FES hand therapy self-managed by sub-acute SCI participants and their caregivers: a usability study. J Neuroeng Rehabil. 2021;18(1):1–18. 33632262 10.1186/s12984-021-00838-y PMC7905902

[53] Blain-Moraes S, et al. Barriers to and mediators of brain-computer interface user acceptance: Focus group findings. Ergonomics. 2012;55(5):516–25. 22455595 10.1080/00140139.2012.661082

[54] LaHue SC, et al. Parkinson’s disease patient preference and experience with various methods of DBS lead placement. Parkinsonism Relat Disord. 2017;41:25–30. 28615151 10.1016/j.parkreldis.2017.04.010

[55] Lewis CJ, et al. The impact of subthalamic deep brain stimulation on caregivers of Parkinson’s disease patients: an exploratory study. J Neurol. 2015;262(2):337–45. 25381461 10.1007/s00415-014-7571-9

[56] Cabrera LY, et al. Beyond the cuckoo’s nest: patient and public attitudes about Psychiatric Electroceutical interventions. Psychiatr Q. 2021;92(4):1425–38. 33864542 10.1007/s11126-021-09916-9 PMC8531080

[57] Jongsma KR, Bredenoord AL. Ethics parallel research: an approach for (early) ethical guidance of biomedical innovation. BMC Med Ethics. 2020;21(1):1–9. 32867753 10.1186/s12910-020-00524-z PMC7461257

[58] Lozano AM, et al. Deep brain stimulation: current challenges and future directions. Nat Reviews Neurol. 2019;15(3):148–60. 10.1038/s41582-018-0128-2 PMC6397644 30683913

[59] Schuepbach W, et al. Neurostimulation for Parkinson’s disease with early motor complications. N Engl J Med. 2013;368(7):610–22. 23406026 10.1056/NEJMoa1205158

[60] Follett KA, et al. Pallidal versus subthalamic deep-brain stimulation for Parkinson’s disease. N Engl J Med. 2010;362(22):2077–91. 20519680 10.1056/NEJMoa0907083

[61] Mahajan A, et al. Global variability in Deep Brain Stimulation practices for Parkinson’s Disease. Front Hum Neurosci. 2021;15:667035. 33867961 10.3389/fnhum.2021.667035 PMC8044366

[62] Bublitz C, Gilbert F, Soekadar SR. Concerns with the promotion of deep brain stimulation for obsessive-compulsive disorder. Nat Med. 2023. 10.1038/s41591-022-02087-5 36604537

[63] White P, Rickards H, Zeman A. Time to end the distinction between mental and neurological illnesses. BMJ. 2012;344. 10.1136/bmj.e3454 22628005

[64] Martin JB. The integration of neurology, psychiatry, and neuroscience in the 21st century. Am J Psychiatry. 2002;159(5):695–704. 11986119 10.1176/appi.ajp.159.5.695

[65] Ferretti A, Ienca M. Enhanced cognition, enhanced self? On neuroenhancement and subjectivity. J Cogn Enhancement. 2018;2(4):348–55. 10.1007/s41465-018-0109-9

[66] Montemayor J, et al. Deep brain stimulation for Parkinson’s Disease: why earlier use makes Shared decision making important. Neuroethics. 2022;15(2):1–11. 10.1007/s12152-022-09496-w

[67] Maier F, et al. Subjective perceived outcome of subthalamic deep brain stimulation in Parkinson’s disease one year after surgery. Parkinsonism Relat Disord. 2016;24:41–7. 26827110 10.1016/j.parkreldis.2016.01.019

